# Associations Among in-The-Moment Emotional Clarity, Emotion Regulation, and Psychopathology in Obsessive-Compulsive Disorder

**DOI:** 10.1155/da/7799020

**Published:** 2025-11-14

**Authors:** Nicola Hohensee, Claudia Bischof, Fanny Alexandra Dietel, Nadja Klein, Philipp Doebler, Ulrike Buhlmann

**Affiliations:** ^1^Christoph-Dornier Foundation Münster, Schorlemerstraße 26, Münster 48143, Germany; ^2^Department of Psychology and Sports Sciences, University of Münster, Fliednerstraße 21, Münster 48149, Germany; ^3^Department of Psychology, University of Osnabrück, Artilleriestraße 34, Osnabrück 49076, Germany; ^4^Scientific Computing Center, Karlsruhe Institute of Technology, Karlsruhe 76131, Germany; ^5^Department of Statistics, TU Dortmund University, Dortmund 44221, Germany

**Keywords:** ecological momentary assessment, emotion regulation, emotional clarity, longitudinal study, multilevel models, OCD

## Abstract

Past research showed that lower emotional clarity (EC) was associated with more maladaptive emotion regulation (ER) and psychopathology, such as obsessive-compulsive (OC) disorder (OCD). However, most of these studies used single time-point, retrospective self-reports. Next to high risk for recall biases and low ecological validity, this assessment method is only able to capture between-person differences (i.e., individuals generally high vs. low in EC). It therefore neglects temporal variations in EC and resulting within-person differences (i.e., moments with higher-than-usual vs. lower-than-usual EC within one individual). To address this gap, our study uses intensive longitudinal data based on a 6-day ecological momentary assessment (EMA) design with up to six measurements daily. In total, *N* = 72 individuals diagnosed with OCD and *N* = 54 mentally healthy controls (HCs) reported on EC, ER behavior, and OC symptoms. Our results confirm that EC was significantly lower in individuals with OCD, even when controlling for baseline depression. Furthermore, lower within-person EC was associated with a higher number of used avoidance-oriented ER strategies, a lower number of engagement-oriented ER strategies and lower ER effectiveness. Surprisingly, these associations were more pronounced in the control (vs. OCD) group. In individuals with OCD, results indicated a negative concurrent (but not subsequent) association between EC and OC symptoms. Explanations for nonsignificant findings and possible implications for the role of EC in OCD are discussed.

## 1. Introduction

Emotional intelligence is defined as a set of abilities including the perception, understanding, and management of emotions [1]. Emotional clarity (EC), that is, an individual's meta-knowledge about their own affective experience, is considered to be a core feature of emotional intelligence [1–5]. It is theorized that the subjective certainty about which emotions one is feeling in a certain situation can facilitate appropriate judgement about adaptive regulatory behavior [6, 7]. In line with these theoretical claims, lower EC was associated with more repetitive negative thinking and use of putatively maladaptive emotion regulation (ER) strategies (e.g., emotional suppression) as well as lower perceived success of regulatory efforts in past studies [8–11].

The link between EC and ER is also relevant from a clinical perspective: habitual use of avoidance-oriented ER strategies is quite consistently linked to a detrimental emotional outcome and different psychopathologies in the long run, which is why they are referred to as “putatively maladaptive.” On the other hand, more engagement-oriented ER strategies (e.g., reappraisal) are considered to be “putatively adaptive” because they are more consistently linked to beneficial long-term outcomes [12]. 1 While it is generally assumed that using a higher number of strategies to regulate emotions is beneficial [13], the simultaneous use of a higher number of avoidance-oriented strategies seems to worsen emotional outcomes and vice versa [14, 15]. Importantly, past research also established direct associations between psychopathology and EC: clinical populations reported lower scores in comparison to healthy controls [16–19]. In addition, cross-sectional and longitudinal studies showed that lower EC was associated with higher symptom severity and emotional well-being in general, that is, higher negative and lower positive affect [10, 20–24].

However, the vast majority of research conceptualizes EC as a stable trait assessed via single time-point, retrospective self-reports. These measures focus on the extent to which an individual typically understands their emotions as well as resulting between-person differences (i.e., individuals with high vs. low EC). Recent studies show; however, that clarity about one's own emotions can vary across time and within different situations depending on the specific context [25]. Therefore, it is important to additionally consider the state component of EC, defined as the extent to which an individual understands their emotions in a specific moment. Substantial situational and temporal variation necessarily result in within-person differences (i.e., moments with higher-than-usual vs. lower-than-usual EC within one individual) which might then lead to differential short-term outcomes. Importantly, existing theoretical frameworks imply exactly these within-person associations. That is, when an individual experiences higher EC, this person should be able to use more adaptive regulatory behavior. We; however, cannot fully evaluate these theoretical claims using single time-points. Especially because between-person and within-person associations do not necessarily align [26], it is important to implement study designs that are able to tease apart both components. One promising approach are intensive longitudinal study designs, that is, daily diaries or ecological momentary assessment (EMA). Using a daily diary design, Eckland and Berenbaum [27] showed that higher-than-usual, daily EC was associated with less repetitive negative thinking and more active problem solving in college students. Hohensee et al. [9] found consistent associations between higher daily EC and less use of several putatively maladaptive ER strategies (i.e., rumination, dampening, emotional suppression, behavioral avoidance) in youth. In addition, higher current emotional intelligence was associated with less disordered eating at the same and subsequent time-point in college students in an EMA design [28]. Thus, first studies point towards within-person associations that are in line with past between-person findings.

Another relevant advantage of intensive longitudinal designs is the reduction of recall biases. This might be especially fruitful in clinical populations, because they are more prone to the biased perception of their own emotional experiences and regulatory abilities [29]. In line with this assumption, Park and Naragon-Gainey [30] found that higher current EC was associated with less subsequent internalizing symptoms via successful ER in treatment-seeking individuals. Interestingly, they also found evidence for the reverse relation, that is, internalizing symptoms worsening subsequent EC, indicating a “vicious circle” of deficient emotional abilities and psychopathology. But studies on current EC in clinical (instead of subclinical) populations are limited, which are essential to increase our understanding of daily-life mechanisms and thus, potential target points for future interventions.

One clinical population that is known to have especially large difficulties to assess internal states, including emotions, are individuals with obsessive-compulsive (OC) disorder (OCD) [31]. OCD is a mental disorder characterized by intrusive thoughts, that is, obsessions, and repetitive behaviors, that is, compulsions, that are supposed to reduce emotional distress caused by obsessions [32]. It is a chronic and highly impairing disorder [33], which is also illustrated by improvable remission rates around 45% after cognitive-behavioral therapy [34]. Furthermore, drop-out rates from treatment are still too high with around 19% [35]. Given this burden, it appears paramount to enhance further understanding of the etiology and maintenance of OCD to improve treatment outcomes on the long run. In this regard, Dar et al. [36] recently developed the seeking proxies for internal states (SPIS) model for OCD. The SPIS model assumes that difficulties to assess internal states, e.g., emotions, lead to doubts and ambiguity which individuals with OCD try to reduce by seeking proxies for these internal states, such as fixed rules and rituals [36]. Indeed, initial evidence indicates that lack of EC might be one promising factor contributing to OC symptoms. Specifically, individuals with OCD, versus clinical and nonclinical controls, reported higher levels of alexithymia and lower levels of emotional intelligence [23, 37, 38]. Cross-sectionally, OC symptom severity was negatively associated with EC [39, 40]. Importantly, Bredemeier et al. [41] showed that lack of EC measured at baseline predicted higher OC symptom severity at post-treatment in patients with OCD. Taken together, first cross-sectional and longitudinal studies suggest between-person associations between EC and OC symptoms, in a single time-point and several months apart. Studies using intensive longitudinal designs and assessing current EC as well as its (bidirectional) within-person associations with psychopathology in OCD are, however, still missing. In this regard, it also remains unclear in which short-term time scale mechanisms connecting EC and OC symptoms, e.g., as described in the SPIS model, unfold in daily life: Are within-person associations detectable immediately within concurrent time points or do associations need a few hours to unfold? Insight into these questions would be informative regarding the degree of automaticity of these processes and the potential clinical impact of EC in daily life of individuals with OCD. Relatedly, it remains open whether associations between maladaptive ER patterns and EC are even stronger in individuals with (vs. without) OCD. Following the SPIS model and the assumed within-person associations, low EC in OCD (vs. mentally healthy controls [HCs]) might lead to even higher maladaptive efforts to regulate emotions and reliance on these maladaptive patterns as “proxies,” indicated by a higher number of used avoidance-oriented ER strategies and lower perceived ER success.

Taken together, the current study addresses an important gap in the literature. To our knowledge, we are the first assessing current EC and its associations with regulatory behavior and psychopathological outcomes in clinical OCD using a 6-day EMA design. The use of EMA designs in clinical populations is especially important for three reasons. First, we need to increase our understanding of in-the-moment factors contributing to psychopathology unfolding in daily life to effectively improve treatment options. In this regard, it is crucial to tease apart between-person and within-person associations of EC, as well as the time scale of within-person processes. Second, we should clarify possible bidirectional in-the-moment associations with psychopathology to be more aware of possible negative feedback loops. Third, clinical populations, and especially individuals with OCD, have large difficulties to assess internal states [31]. Thus, the assessment of emotional abilities and experiences with retrospective, trait-based self-reports might be especially biased.

Based on the literature, we tested the following hypotheses (preregistered hypotheses are indicated with a *⁣*^*∗*^):

  H1*⁣*^*∗*^. Individuals with OCD will report lower current EC than HCs across all assessment points.
  H2a. Lower EC will be associated with less self-perceived ER effectiveness (between-person and within-person level) across all participants, irrespective of group status*⁣*^*∗*^. This association should be especially pronounced in individuals with OCD (vs. HC).
  H2b. Lower EC will be associated with (1) a higher number of used avoidance-oriented ER strategies and (2) a lower number of used engagement-oriented ER strategies (between-person and within-person level) across all participants, irrespective of group status. These associations should be especially pronounced in individuals with OCD (vs. HC).
  H3. For individuals with OCD, lower EC will be associated with a higher likelihood for the presence of (1) current and (2*⁣*^*∗*^) subsequent OC symptoms. Vice versa, the presence of OC symptoms will be associated with lower (1) current and (2*⁣*^*∗*^) subsequent EC (between-person and within-person level).

## 2. Method

The present study is part of a larger project examining ER in OCD ([42] provides the main outcome paper).

### 2.1. Transparency and Openness

The study design was preregistered on osf.io in August 2021 before data analysis. Hypotheses and analysis plan were partly preregistered (indicated using an *⁣*^*∗*^ above) with the registration ID osf.io/9b8hk. Due to our study design, participants only reported selectively on regulatory effectiveness and OC symptoms. To maintain a statistical power of at least 80%, we deviated from our preregistration and refrained from analyzing ER effectiveness as mediator for the association between EC and OC symptoms.

Approval for the study was granted by the ethics committee of the Department of Psychology and Sports Science at the University of Münster and we complied with APA ethical standards in the treatment of our sample. All study materials, data, and analysis code are accessible online at osf.io/fr5b4.

### 2.2. Participants

Data were collected from February 2021 to April 2022 with recruitment beginning in January 2021. Upon securing written informed consent, a total of *N* = 155 participants (*n* = 92 individuals diagnosed with OCD and *n* = 63 HCs) were invited for an initial diagnostic session. All participants had to be between 18 and 65 years old and fluent in German. Additionally, individuals with OCD had to meet diagnostic criteria as outlined in the DSM-5 [32] at the time of testing. They were excluded when they had a lifetime diagnosis of psychosis, bipolar disorder, or borderline personality disorder, recent substance abuse within the last 5 years, alterations in psychotropic medication within the past 8 weeks, and current suicidality. Controls were excluded if they had ever received a diagnosis of a mental disorder, undergone psychotherapy, or taken psychotropic medication. Consequently, *n* = 1 individual in the control group and *n* = 8 individuals with OCD were excluded during the initial diagnostic session because their answers in the interview diverged from answers during the telephone screening so that inclusion criteria were no longer met. In the OCD group, *n* = 11 individuals did not show up for the initial diagnostic session and *n* = 1 participant had to be excluded due to technical issues. In the control group, *n* = 7 individuals did not show up for baseline assessments while also *n* = 1 participant was excluded because of technical issues. Thus, in total the sample included *N* = 72 participants with OCD and *N* = 54 mentally healthy individuals. For the analysis of hypothesis H2, an additional *n* = 15 individuals were excluded due to missingness in ER data, that is, these participants did not report at any EMA time point that they actively tried to change their emotions (below is description of our branched EMA design). Therefore, the final sample size included *N* = 111 for hypotheses H2a and H2b. Most of the participants with missing ER data were HCs (*n* = 11), they reported significantly lower baseline anxiety symptoms (*F* [1124] = 7.181, *p*=0.008), stress symptoms (*F* [1124] = 3.918, *p*=0.050), and OC symptoms (*F* [1124] = 6.505, *p*=0.012). Otherwise, they did not significantly differ from the rest of the sample in gender distribution, age, or baseline depressive symptoms (all *p* > 0.05). Demographics for the full sample of *N* = 126 individuals were the following: the average age of individuals in the OCD group was 29.17 years (SD = 8.04 years), while controls reported an average age of 27.76 years (SD = 4.59 years). Gender distribution was similar across groups, with 79.17% of the OCD group and 79.63% of the controls identifying as female. No significant group differences were observed regarding age or gender (*p* > 0.05). Further sociodemographic and clinical characteristics are presented in Table 1.

### 2.3. Power Analysis

The sample size was determined based on an a priori power analysis conducted for the main outcome paper ([42] provides further details). We used simulations with the R package simr (version 1.0.7; [43]). To attain a statistical power of at least 80% for analyses involving both groups, a recommended sample size of *N* = 100 was predetermined. For analyses only including the OCD group, the suggested sample size was *N* = 70.

## 3. Materials

For a detailed description of all materials and study procedures, see [42].

### 3.1. Baseline Assessment

For the current study, baseline measures were only used for validation of EMA measures and to ensure inclusion and exclusion criteria.

#### 3.1.1. Diagnostic Interview for Mental Disorders ([DIPS] [44])

To assess OCD diagnoses, we used the structured clinical interview for mental disorders based on DSM-5 criteria. Interrater reliability was based on 20% of randomly selected ratings for the OCD section conducted by Nicola Hohensee and Claudia Bischof. During data collection, they were both in the advanced stages of their training as cognitive-behavioral psychotherapists. Interrater reliability was excellent (Cohen's *κ* 1).

#### 3.1.2. Yale-Brown OC Scale ([Y-BOCS] [45, 46])

To assess OCD symptom severity, we used the Y-BOCS, a 12-item semi-structured interview. Based on randomly drawn 20% of the conducted interviews, the intraclass correlation coefficient (ICC) of 0.99 indicated excellent agreement between the two raters.

#### 3.1.3. Depression Anxiety Stress Scales ([DASS-21] [47, 48])

The DASS-21 is a self-report questionnaire with 21 items assessing the severity of depression, anxiety, and stress symptoms on three subscales. Internal consistency in the current sample was high, with *α* = 0.92 for the depression subscore, *α* = 0.87 for the anxiety subscore and *α* = 0.91 for the stress subscore.

#### 3.1.4. Difficulties in ER Scale ([DERS] [49, 50])

The DERS is a 36-item, self-report questionnaire that assesses problems in understanding and accepting emotions, engaging in goal-directed behavior, and accessing effective ER strategies. The internal consistency for the DERS total score in the current sample was very high (*α* = 0.96).

#### 3.1.5. Maladaptive and Adaptive Coping Style Questionnaire ([MAX] [51])

The MAX is a 21-item, self report questionnaire examining coping profiles in response to distress. The internal consistency in the current sample was good, with *α* = 0.89 for the adaptive coping subscale, with *α* = 0.86 for the maladaptive coping subscale, and *α* = 0.59 for the avoidance subscale.

### 3.2. EMA Assessment

For the current study, the following EMA measures were used to test all hypotheses of interest. Descriptive statistics for all EMA measures can be found in Table 2.

#### 3.2.1. Current Negative Affect

Current negative affect was measured on a five-point Likert scale from 1 (not at all) to 5 (extremely) and mostly based on the emotion sense application [52]. It comprised seven items and was augmented by OCD-specific emotions (i.e., angry, anxious, lonely, sad, ashamed, guilty, disgusted). Current negative affect was operationalized as the mean of these items. The disgust item was added after the first 10 participants had been assessed, based on participant feedback that this disorder-specific emotion was missing. 2 The ICC, as derived from a null model, was 0.61, indicating that 61% of the variance in negative affect was accounted for by between-person variation. Due to between-group heteroscedasticity, individual ICCs were calculated based on the ratio of the between-person variance (across the two groups) and the total variance. The total variance was derived from the sum of the between-person variance and the person-specific residual variances using multilevel Gaussian location-scale models (Data Analysis Section 2.6 provides further details). The range of ICCs was 0.52–0.78.

#### 3.2.2. Current EC

Participants were asked how clearly they were able to identify the emotions they were feeling in the respective moment using a five-point Likert scale from 1 (not clearly at all) to 5 (very clearly) adapted from Park and Naragon-Gainey [30]. The ICC was 0.55 and when considering between-group heteroscedasticity, the range of individual ICCs was 0.38–0.91. The validity of our item was ensured by a significant, negative correlation with the “Lack of EC” DERS subscale (*r* = −0.60, *p* < 0.001).

#### 3.2.3. ER Strategies and Perceived Effectiveness

Participants were asked whether they tried to change how they felt. If yes, they were presented with a multiple-selection list of nine specific ER strategies, categorized as either avoidance-oriented (i.e., emotional suppression, distraction, expressive suppression, rumination, behavioral avoidance) or engagement-oriented (i.e., problem-solving, cognitive reappraisal, introspection, seeking advice). The current use of strategies was measured as the total number of avoidance- or engagement-oriented strategies selected at each time point [13, 53, 54]. The ICC was 0.47 for avoidance-oriented ER strategies and 0.39 for engagement-oriented strategies. Considering between-group heteroscedasticity, the range of individual ICCs was 0.42–0.85 for avoidance-oriented ER strategies and 0.36–0.67 for engagement-oriented ER strategies. Regarding the validity of our EMA variables, we could show significant positive correlations between the maladaptive coping subscale of the MAX and the number of avoidance-oriented ER strategies (*r* = 0.27, *p*=0.004) as well as the adaptive coping subscale of the MAX and the number of engagement-oriented ER strategies (*r* = 0.28, *p*=0.003).

Following Daniel et al. [53], participants were asked to evaluate how much better they felt after using the chosen strategies on a scale ranging from 0 (much worse) to 100 (much better). The ICC for ER effectiveness was 0.32. Considering heteroscedasticity, the individual ICCs ranged from 0.30 to 0.41. Again, the validity of our EMA measure was ensured by a significant, negative correlation with the “Lack of effective ER strategies” DERS subscale (*r* = −0.39, *p* < 0.001) and the “Difficulties in goal-directed behavior” DERS subscale (*r* = −0.35, *p* < 0.001).

#### 3.2.4. Current OC Symptoms

Participants in the OCD group reported whether or not they were experiencing obsessions or compulsions separately, at each time point. If yes, they were also asked to rate the intensity of obsessions and compulsions separately on a five-point Likert scale from 1 (mild) to 5 (extreme). Diverging from our preregistration, we decided to use a dichotomous variable indicating the presence of OC symptoms, i.e., obsessions or compulsions or both (instead of intensity averaged across both symptom items) at the current assessment point in our statistical analyses. The reason was as follows: individuals in the OCD group only reported OC symptoms in 935 out of 2277 total observations and, by using a dichotomous variable, we were able to include all possible observations in our analyses (instead of observations where participants rated the intensity of OC symptoms only). The average frequency of OC symptoms reported across the whole EMA period correlated significantly with the Y-BOCS total score (*r* = 0.43, *p* < 0.001), indicating the validity of our chosen EMA measure.

### 3.3. Study Procedure

Data were collected online via video appointments using RED connect software [55], self-report questionnaires using EFS survey [56], and app-based EMA using MovisensXS [57].

After the initial diagnostic session and a briefing for the use of the application, individuals participated on their own smartphones. They were prompted to complete up to 36 EMA prompts within a 6-day period, between 9:00 am and 9:00 pm each, with a minimum 1 h interval between two assessments. Participants had 15 min to respond to incoming alarms and could postpone alarms by 5 or 10 min if needed. Additionally, they had the option to temporarily pause the application to ensure uninterrupted focus during important engagements. All EMAs were answered based on the participant's current state just prior to receiving the alarm. Participants were compensated up to 80€, with an additional potential bonus of 20€ if they completed at least 80% of the assessments.

### 3.4. Data Analysis

The data were analyzed using R [58]. We used chi-square tests and one-way ANOVAs for group comparisons. Spaghetti plots were used to determine the presence of temporal patterns across time points. Because we could not observe significant trends of reactivity for any variables of interest, we refrained from including day of assessment as control variable in our multilevel models.

To test our hypotheses, we conducted multilevel regression models which considered the hierarchical structure of our EMA data. To address between-group heteroscedasticity, multilevel Gaussian location scale models, implemented in the gauss() function from the R package mgcv (version 1.8–33; [59]), were used. This approach allows modeling the precision parameter of the response as a function of predictors (here group membership, gender, and age). This results in individual-specific residual variances which reduce the risk of overly conservative or liberal inference due to heteroscedasticity. Incorporated within a (penalized) likelihood framework, standard errors are obtained based on the posterior distribution of the model coefficients [59, 60], and we used them to compute 95% confidence intervals (CIs) for all relevant quantities. Random effect structure of the multilevel models was determined in two steps: first, because measurement points were nested within days within participants, we tested whether a two-level (i.e., measurements nested within participants) or a three-level (i.e., measurements nested within days nested within participants) model was justified by computing ICCs. Due to between-group heteroscedasticity, we computed individual ICCs based on the ratio of the between-person variance (across the two groups) and the total variance. The total variance was derived from the sum of the between-person variance and the person/day-specific residual variances using multilevel Gaussian location-scale models. Only if the median of these person/day-specific ICCs was larger than 0.01 (i.e., differences between days explaining more than 1% of variance), we included an additional nested random intercept in the model [61]. Second, regarding random slopes, we used model comparisons of AIC values, which is preferred over generalized likelihood ratio test for generalized additive models [59]. We chose more complex models over basic models that just included random intercepts if the AIC value was more than 10 units lower [62]. For the specific random effects structure of each model, please refer to Table 3. 3 To test the specific hypotheses, the multilevel regression models were adapted as follows (exact equations for all models can be found in the supplements).

To test hypothesis H1, current EC was predicted from the level-2 predictors group status (OCD vs. HC), baseline depression score, age, and gender.

To investigate hypotheses H2a and H2b, current (1) ER effectiveness, (2) number of used avoidance-oriented ER strategies, and (3) number of used engagement-oriented ER strategies^4^ were predicted from current, person-mean centered EC, group membership, and an interaction effect between current EC and group. In addition, we assessed between-person associations by including the person mean of EC (which was grand-mean centered) and an interaction effect between mean EC and group. All models were controlled for current person-mean centered negative affect, age, and gender. Diverging from our preregistration, we did not include the score of the dependent variable from the previous assessment point (i.e., the lagged outcome variable) as a control variable in our models. Since ER behavior was only assessed when participants indicated that they tried to change their emotions, the score of the lagged outcome variable was often missing. Therefore, including this control variable would have reduced our total number of observations from *N* = 920 to *N* = 277. To maintain statistical power, we therefore refrained from using this control variable. Full models are still reported in Table A.1 of the supporting information.

To test hypothesis H3, we predicted (1) current presence of OC symptoms (binary variable) from person-mean centered current EC and (2) current EC from current presence of OC symptoms. Additionally, all models included the grand-mean centered person mean of the predictor variable (i.e., EC or presence of OC symptoms) and were controlled for the lagged outcome variable, age, and gender. Second, we predicted (1) subsequent presence of OC symptoms (binary variable) from person-mean centered current EC and (2) EC at the subsequent time point from current presence of OC symptoms. Again, all models included the grand-mean centered person mean of the predictor variable (i.e., EC or presence of OC symptoms) and were controlled for the lagged outcome variable, age, and gender.

## 4. Results

### 4.1. Descriptive Analysis

Overall, participants showed a high compliance rate with an average 89.7% of completed questionnaires in total (i.e., 32.3 [*SD* = 4.23] questionnaires). In total, 4063 observations were included in our analyses assessing group differences in EC. Because the use of ER strategies as well as ER effectiveness were only assessed when participants indicated having tried to change their emotions (i.e., in around 23% of the assessments), multilevel models conducted for hypotheses H2a and H2b included 920 observations. For multilevel models assessing the association between EC and presence of OC symptoms within the OCD group, we ended up with 1841 observations. A Pearson correlation matrix of all variables of interest can be found in Table C.1 of the supporting information.

### 4.2. Hypothesis Testing

Estimates for all multilevel models are presented in Table 3.

#### 4.2.1. Group Differences in EC (Hypothesis H1)

As expected, the main effect of group was significant, indicating that individuals with OCD reported significantly lower EC than controls. Group differences remained significant when controlling for grand-mean centered score of the DASS-21 depression subscale, age, and gender.

#### 4.2.2. Associations Between ER Effectiveness and EC (Hypothesis H2a)

**Figure 1:**
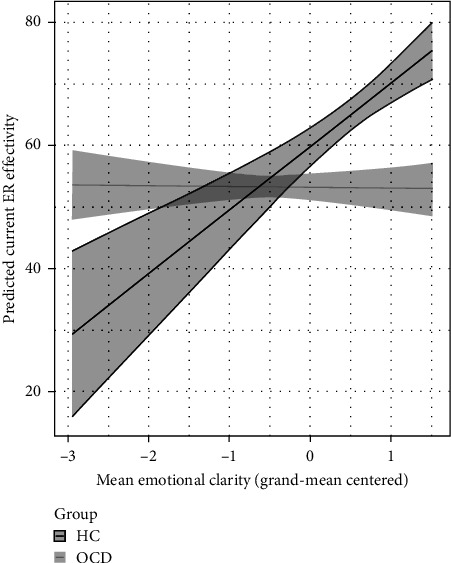
Plot for the significant interaction effect between mean emotional clarity and group membership when predicting self-perceived ER effectiveness. ER, emotion regulation; HC, mentally healthy controls; OCD, individuals with obsessive-compulsive disorder.

For the between-person level, as expected, we found a significant main effect of EC, indicating that individuals with generally higher (vs. lower) EC reported higher ER effectiveness in daily life. In addition, the interaction between mean EC and group membership was significant. Surprisingly, the positive association between mean EC and current ER effectiveness was present in controls (but not in individuals with OCD; Figure 1), when setting all other variables to their mean.

For the within-person level, in line with our hypothesis, we found a significant main effect of EC again. On assessment points with a higher-than-usual (vs. lower-than-usual) EC, individuals reported higher ER effectiveness. The interaction effect between EC and group was not significant. Results remained unchanged when controlling for current negative affect, age, and gender.

#### 4.2.3. Associations Between ER Strategies and EC (Hypothesis H2b)

**Figure 2:**
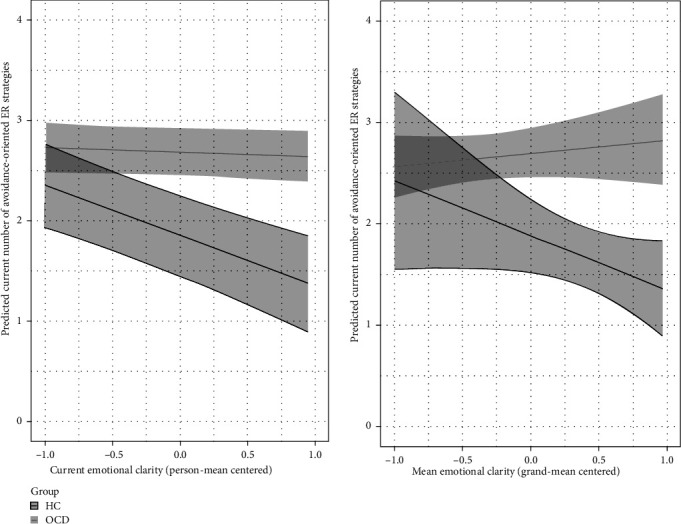
Plot for the significant interaction effect between current emotional clarity and group membership when predicting currently used number of avoidance-oriented ER strategies. ER, emotion regulation; HC, mentally healthy controls; OCD, individuals with obsessive-compulsive disorder.

For the between-person level, surprisingly, the main effect of EC was nonsignificant for both, the number of avoidance-oriented and engagement-oriented ER strategies. The interaction between mean EC and group was nonsignificant for engagement-oriented ER strategies. For avoidance-oriented strategies, group membership emerged as a significant moderator for the association with mean EC. Surprisingly, controls (but not individuals with OCD) who reported generally higher (vs. lower) EC, reported a lower number of used avoidance-oriented ER strategies in daily life (Figure 2, right plot).

For the within-person level, in line with our hypothesis, the main effect of current EC was significant for the currently used number of avoidance-oriented and engagement-oriented ER strategies. On assessment points with higher-than-usual (vs. lower-than-usual) EC individuals reported a lower number of used avoidance-oriented ER strategies. On assessment points with higher-than-usual (vs. lower-than-usual) EC individuals reported a higher number of used engagement-oriented ER strategies. The interaction effect between current EC and group was only significant for avoidance-oriented but not engagement-oriented ER strategies. Not in line with our hypotheses, the association between EC and ER strategies was again stronger in controls (vs. individuals with OCD), when setting all other variables to their mean (Figure 2, left plot). Most results remained unchanged when controlling for current negative affect, age, and gender. The only exceptions were the interaction between mean EC and group for avoidance-oriented ER strategies (which turned nonsignificant) and the interaction between current EC and group for engagement-oriented ER strategies (which turned significant). We present an interaction plot of the latter in Figure D.1 of the supporting Information.

#### 4.2.4. Associations Between Presence of OC Symptoms and EC (Hypothesis H3)

For the between-person level, neither the main effect of mean EC (when predicting the presence of OC symptoms) nor the main effect of present OC symptoms (when predicting EC) were significant, which was not in line with our hypotheses.

For the within-person level, as expected, the main effect of current EC was significant when predicting the current presence of OC symptoms, indicating that on assessment points with higher (vs. lower) EC, individuals were less likely to report OC symptoms. On the other hand, the main effect of the current presence of OC symptoms was significant when also predicting current EC. Thus, as expected, on assessment points where individuals reported OC symptoms (vs., no OC symptoms), they reported lower EC. Results in both models remained significant when controlling for the score of the outcome variable from the previous assessment point, age, and gender.

For models predicting EC or the presence of OC symptoms on the subsequent assessment point, surprisingly, all results were nonsignificant.

## 5. Discussion

The present study is one of the first to examine in-the-moment associations among EC, ER behavior, and psychopathology in a large sample of individuals diagnosed with OCD. By additionally including a group of HCs, we were also able to investigate differences in EC and differential effects on regulatory behavior. Extending prior work, we showed between-person and within-person associations between EC, ER behavior, and psychopathology, but these relations were partly moderated by clinical status. Overall, three major findings emerged that will be discussed in more detail.

### 5.1. Differences in EC and its Associations With Regulatory Behavior in Individuals With OCD Versus Controls

EC was significantly lower in individuals diagnosed with OCD (vs. HC), even after controlling for baseline depression scores, age, and gender. This finding is in line with past research and the recently developed SPIS model postulating the central role of restricted access to internal states in OCD [23, 37, 38]. Clinical status also partly moderated the association between EC and ER behavior, but in the opposite direction of our hypotheses. On the between-person level, higher (vs. lower) EC was associated with higher ER effectiveness and a lower number of used avoidance-oriented ER strategies in controls (but less so individuals with OCD)^5^. Similarly, higher-than-usual EC was associated with a lower number of currently used avoidance-oriented ER strategies in controls on the within-person level. These findings appear surprising because they may indicate that EC is less important for ER behavior in individuals with OCD, contrary to theoretical assumptions in the SPIS model. One possible explanation might be; however, that “true” diminished access to one's emotional experiences gets mixed up with reduced confidence in own emotional abilities when self-reports are used to assess EC in OCD. Past research already established that confidence in cognitive processes is more impaired in OCD than actual performance, which might be true for emotional processes as well [63]. The dilemma of using self-reports when assessing psychological constructs that are, by definition, characterized by a certain lack of insight has been discussed in the past (for alexithymia see [18]). Therefore, future work is needed to investigate the relation between EC and ER in OCD using indirect measures (e.g., response times to affect items; [64]) or observer rated EC (e.g., [18] for alexithymia). Another possible explanation might be that we did not sufficiently consider contextual variables in the relation between EC and ER behavior. The SPIS model emphasizes the reliance on fixed rules and norms to substitute limited emotional access. Thus, lack of EC might be more strongly associated with generally rigid ER patterns instead of the pronounced use of certain regulatory strategies that are “maladaptive” by default. Recent research is emphasizing an individual's ability to monitor and modify ER strategies depending on the situational demands and personal goals, that is, ER flexibility [65]. In this regard, it will be essential to consider more situational characteristics in future EMA studies. It could be the case that the perception of certain situational characteristics (e.g., controllability, adversity) and ER goals (e.g., up- and downregulation of negative affect) differ systematically between clinical and nonclinical populations. Individual differences might, in turn, require different patterns of ER strategies which might even lead to the current upregulation of negative affect in order to accomplish an important personal goal. The beneficial effects of higher EC could therefore be masked when we only focus on the use of presumably maladaptive regulatory strategies on one hand or mere gain of current emotional-wellbeing on the other in OCD.

### 5.2. EC Is Associated With Presumably More Adaptive ER Behavior

In line with our hypotheses, individuals with generally higher (vs. lower) EC reported higher ER effectiveness in daily life. Similarly, results on the within-person level were consistent with our hypotheses: on assessment points with higher-than-usual EC, individuals reported higher current ER effectiveness, a higher number of engagement-oriented ER strategies, and a lower number of avoidance-oriented ER strategies. These findings are in line with previous research using intensive longitudinal designs indicating that higher current EC can facilitate adaptive choices in regulatory behavior [9, 27, 30]. It also emphasizes the relative importance of EC for benefits in within-person ER processes in contrast to gains via between-person differences in EC. We also extended previous findings by looking at a broader range of criteria for presumably “adaptive” ER behavior (i.e., self-perceived ER effectiveness, used number of avoidance-oriented, engagement-oriented ER strategies). In addition, we compared associations between EC and ER in a clinical population diagnosed with OCD and a control group of mentally healthy individuals. Thus, even though group membership partly moderated these associations, EC seems to play an important role in clinical and nonclinical populations, especially for the use of engagement-oriented ER strategies (which were not moderated by group). Future research should continue to use intensive longitudinal designs in order to further investigate context factors of in-the-moment ER processes.

### 5.3. Concurrent (but Not Subsequent) Associations Between EC and OC Symptoms

In individuals with OCD, OC symptoms were less likely to be present when EC at the current assessment point was higher-than-usual. This finding is in line with previous cross-sectional studies in OCD and the SPIS model [36, 39, 40]. Our study; however, extends past OCD research in several ways. First, we also showed that, vice versa, current EC was higher when OC symptoms were less likely to be present as well. Because we used an intensive longitudinal design, we were able to control our findings for auto-correlation (i.e., EC or presence of OC symptoms one to two hours earlier). In addition, our study design enabled us to assess concurrent and subsequent within-person associations in daily life. Surprisingly, associations with subsequent outcomes were nonsignificant, which was not in line with the SPIS model and past EMA research in other psychopathologies [30, 36]. This may indicate that EC and OC symptoms are only concurrently related in daily life, but do not have a direct impact on each other in the longer-term, a few hours later. Instead, it might be plausible that subsequent associations only emerge when assessing indirect effects, for example, via ER processes [30]. Future studies with higher statistical power should further investigate bidirectional mediation models of EC and OC symptoms with ER processes serving as potential mediators. As mentioned earlier, another potential methodological explanation might be the use of self-reports to assess EC instead of more indirect or performance-based assessment methods. Associations between EC and OC symptoms may be stronger when measures are used that can more clearly differentiate between reduced confidence in one's own emotional abilities and actual lower performance (e.g., [37]). One alternative methodological explanation may be that we were not able to look at OC symptom intensity due to the overall low frequency of reported symptoms. Using a dichotomous outcome (i.e., OC symptoms present yes/no) instead might have limited our chances to detect more fine-grained associations with EC. Therefore, future studies with larger samples or alternative (nonbranched) EMA designs as well as alternative measurement methods for EC are needed.

### 5.4. Strengths and Constraints on Generality

The present study is, to our knowledge, the first one to use an EMA design to assess associations among EC, ER behavior, and psychopathology in a large sample of individuals with OCD and HCs. Critically, EMA designs reduce recall biases, increase ecological validity, and allow us to differentiate pertinent associations on a between-person and on a within-person level. In addition, we assessed a wide range of potential markers for adaptive regulatory behavior (i.e., number of used engagement-oriented and avoidance-oriented strategies, self-perceived regulatory effectiveness), and were thus able to compare their differential associations in a clinical versus nonclinical sample.

However, the current study has some limitations that need to be acknowledged. First, we used a branched EMA design (with up to six assessment points daily) where participants only selectively reported on ER behavior and OC symptoms. This design choice significantly decreased our statistical power and may have contributed to the nonsignificance of some of our results. Relatedly, the temporal resolution of our study design (with up to 2 h between assessment points) may have been too low to detect small-sized associations between EC and OC symptoms at the subsequent assessment point. Even though we wanted to reduce participant's burden, we need future studies with nonbranched EMA designs using a higher temporal resolution to capture ER dynamics even better. Second, we did not include a clinical control group (e.g., with major depression or social anxiety) in our study. Thus, we cannot infer whether the found associations are specific to OCD. Third, the generalizability of our findings is somewhat limited due to sample demography (as previously discussed in [42]). Relatedly, we did not experimentally manipulate EC or OC symptoms which prevents us from drawing any causal conclusions. In addition, the use of avoidance-oriented and engagement-oriented ER strategies may be related, but addressing this question was beyond the statistical power and scope of the current investigation. Future research should use multivariate multilevel models, considering both types of ER strategies simultaneously or assess associations of EC with novel measures combining avoidance-oriented and engagement-oriented ER strategies (e.g., maladaptive ER ratio; [66]). Fourth, our data collection period (February 2021–April 2022) coincided with periods of social isolation due to the COVID-19 pandemic. We tried to address this by only conducting the 6-day EMA when participants were not in quarantine. We also asked participants to inform us if they had to go into quarantine during EMA study participation, which did never happen. We cannot; however, exclude the possibility that other restrictions beyond quarantines had an impact on the emotional well-being of participants which might have biased our results.

## 6. Conclusions

The present study shows that higher EC (especially on the within-person level) is associated with putatively more adaptive ER behavior. In addition, we found a significant association between within-person EC and presence of OC symptoms in both directions, maybe indicating a negative feedback loop. Findings should be replicated and extended in future research using multimethod assessments for EC, nonbranched EMA designs or experiments. If future findings also point towards EC as a potential resilience factor in OCD, EC might be worthwhile to focus more on in therapeutic interventions (e.g., [67]). Partly differential result patterns in individuals with OCD and controls stress the importance to be cautious to transfer findings from nonclinical to clinical populations. Future studies should further investigate putatively adaptive patterns of regulatory behavior in OCD by including more context factors.

## Figures and Tables

**Table 1:** Sociodemographic and clinical characteristics.

Variables	OCD (*n* = 72)	HC (*n* = 54)	*p*-Value
Age (*M* [SD], range)	29.17 (8.04), 19–55	27.76 (4.59), 20–45	0.25
Gender (female)^a^	*n* = 57 (79.17%)	*n* = 43 (79.63%)	>0.99
Years of education (*M* [SD])	17.56 (3.58)	18.75 (3.83)	0.08
Nationality (%)	*n* = 71 German (98.61%)	*n* = 54 German (100.00%)	>0.99
	*n* = 1 Bulgarian (1.39%)		
Current comorbidity (yes)	*n* = 46 (63.89%)	—	—
Number of comorbidities (*M* [SD])	2.0 (1.16)	—	—
OCD-related disorder (%)	*n* = 4 (8.70%)	—	—
Anxiety disorder (%)	*n* = 38 (82.61%)	—	—
PTSD (%)	*n* = 6 (13.04%)	—	—
Psychosomatic disorder (%)	*n* = 5 (10.87%)	—	—
Depressive disorder (%)	*n* = 15 (32.61%)	—	—
Sexual dysfunction (%)	*n* = 3 (6.52%)	—	—
Eating disorder (%)	*n* = 2 (4.35%)	—	—
Sleeping disorder (%)	*n* = 2 (4.35%)	—	—
ADHD (%)	*n* = 1 (2.17%)	—	—
Current psychotherapy (yes)	*n* = 33 (45.83%)	—	—
Current medication (yes)	*n* = 26 (36.11%)	—	—
DASS-depression (*M* [SD])	7.36 (5.03)	1.52 (2.17)	<0.001
Y-BOCS (*M* [SD])	22.21 (5.56)	—	—

*Note:* HCs, mentally healthy controls.

Abbreviations: ADHD, attention deficit hyperactivity disorder; DASS, depression anxiety stress scales; OCD, obsessive-compulsive disorder; PTSD, posttraumatic stress disorder; Y-BOCS, Yale-Brown obsessive-compulsive scale.

^a^Gender was assessed as female, male, and diverse; however, the option diverse was not selected by any individual.

**Table 2:** Descriptive statistics for EMA items.

Variables	OCD (*n* = 72)	HC (*n* = 54)
Positive affect (*M* [SD])	1.80 (0.57)	2.71 (0.50)
Negative affect (*M* [SD])	0.67 (0.59)	0.17 (0.20)
Emotional clarity (*M* [SD])	2.54 (0.76)	3.29 (0.48)
Frequency of OC symptoms (in %, *M* [SD])	40.73 (20.74)	—
Intensity of OC symptoms (*M* [SD])	3.08 (1.02)	—

	**OCD** **(*n* = 68)**	**HC** **(*n* = 43)**

Frequency ER (in %, *M* [SD])	29.64 (19.99)	19.16 (12.34)
Number of engagement-oriented ER strategies (*M* [SD])	1.74 (0.92)	1.98 (0.89)
Number of avoidance-oriented ER strategies (*M* [SD])	2.64 (0.97)	1.76 (1.02)
Effectiveness of ER (*M* [SD])	53.23 (8.48)	63.61 (10.91)

*Note*: HCs, mentally healthy controls. Item scores were first averaged across all assessment points during the 6-day EMA period within each participant and then descriptive statistics were calculated based on person means. Number of participants differs due to the chosen study design where the skipping of items was partially possible (depending on whether participants indicated that they tried to change their emotions or not).

Abbreviations: ER, emotion regulation; OCD, obsessive compulsive disorder.

**Table 3:** Estimates for multilevel regression models.

Variables	Estimate	*z*-Value	*p*-Value	95% CI	Random effects (SD)*⁣*^*∗*^
Group differences in emotional clarity (hypothesis H1)					

A. Current EC (*D* = 57.1%)					
**Intercept**	**3.193**	**32.083**	**<0.001**	**2.997; 3.388**	0.635
**Group**	**−0.576**	**−4.036**	**<0.001**	**−0.856; −0.296**	—
**Baseline depression**	**−0.148**	**−2.077**	**0.038**	**−0.287; −0.008**	—

Associations between emotion regulation behavior and emotional clarity (hypotheses H2a and H2b)					

B. Current ER effectivity (*D* = 49.4%)					
**Intercept**	**62.023**	**38.572**	**<0.001**	**58.871; 65.174**	6.333 | 2.691 (Day)
**Current EC**	**4.941**	**4.136**	**<0.001**	**2.600; 7.283**	—
**Group**	**−6.960**	**−3.651**	**<0.001**	**−10.697; −3.224**	—
Current EC:group	−2.490	−1.825	0.068	−5.165; 0.184	—
**Mean EC**	**10.332**	**5.276**	**<0.001**	**6.494; 14.171**	—
**Mean EC:group**	**−10.495**	**−4.741**	**<0.001**	**−14.835; −6.156**	—
**Current negative affect**	**−7.959**	**−8.302**	**<0.001**	**−9.837; −6.080**	—

C. Current number of AO strategies (*D* = 59.4%)					
**Intercept**	**1.729**	**9.205**	**<0.001**	**1.361; 2.097**	0.841
**Current EC**	**−0.501**	**−4.630**	**<0.001**	**−0.714; −0.289**	—
**Group**	**0.880**	**3.950**	**<0.001**	**0.443; 1.316**	—
**Current EC:group**	**0.457**	**3.814**	**<0.001**	**0.190; 0.662**	—
Mean EC	−0.542	−1.759	0.079	−1.147; 0.062	—
**Mean EC:group^a^**	**0.679**	**1.963**	**0.050**	**0.001; 1.356**	—
**Current negative affect**	**0.453**	**4.738**	**<0.001**	**0.265; 0.640**	0.414

D. Current number of EO strategies (*D* = 51.4%)					
**Intercept**	**1.906**	**11.341**	**<0.001**	**1.576; 2.235**	0.744
**Current EC**	**0.351**	**3.484**	**<0.001**	**0.154; 0.548**	—
Group	−0.063	−0.317	0.751	−0.454; 0.327	—
Current EC:group^b^	−0.218	−1.957	0.050	−0.436; 0.0003	—
Mean EC	0.484	1.756	0.079	−0.056; 1.025	—
Mean EC:group	−0.155	−0.503	0.615	−0.761; 0.450	—
Current negative affect	0.005	0.047	0.963	−0.186; 0.195	0.496

Associations between presence of OC symptoms and emotional clarity (hypothesis H3)					

E. Current OC symptoms (y/n) (*D* = 28.6%)					
Intercept	−0.446	−0.220	0.826	−4.418; 3.527	15.814 | 0.814 (day)
**Current EC**	**−0.273**	**−2.132**	**0.033**	**−0.524; −0.022**	0.728
Lagged OC symptoms (y/n)	−0.086	−0.696	0.486	−0.326; 0.155	—
Mean EC	−0.106	−0.043	0.966	−4.940; 4.729	—

F. Subsequent OC symptoms (y/n) (*D* = 22.2%)					
Intercept	−0.474	−1.436	0.151	−1.121; 0.173	2.482 | 0.659 (Day)
Current EC	0.057	0.775	0.438	−0.088; 0.202	—
Current OC symptoms (y/n)	0.001	0.010	0.992	−0.227; 0.230	—
Mean EC	−0.099	−0.250	0.803	−0.877; 0.679	—

G. Current EC (*D* = 58.9%)	—	—	—	—	—
**Intercept**	**2.358**	**23.611**	**<0.001**	**2.162; 2.554**	0.679 | 0.109 (Day)
**Current OC symptoms (y/n)**	**−0.113**	**−2.384**	**0.017**	**−0.205; −0.020**	0.257
**Lagged EC**	**0.095**	**4.352**	**<0.001**	**0.052; 0.138**	—
Mean likelihood OC symptoms	−0.078	−0.193	0.847	−0.870; 0.714	—

H. Subsequent EC (*D* = 57.5%)					
**Intercept**	**2.167**	**23.252**	**<0.001**	**1.985; 2.350**	0.574 | 0.082 (Day)
Current OC symptoms (y/n)	0.039	1.086	0.278	−0.031; 0.109	—
**Current EC**	**0.128**	**4.440**	**<0.001**	**0.071; 0.184**	0.148
Mean likelihood OC symptoms	0.079	0.204	0.838	−0.684; 0.843	—

*Note. D*, explained deviance. Significant estimates are written in bold font. Including age and gender as control variables did not change the vast majority of result patterns, the herein reported models do not include these two additional control variables and instable effects are marked.

Abbreviations: AO, avoidance-oriented; CI, confidence interval; EC, emotional clarity; EO, engagement-oriented; ER, emotion regulation; OC, obsessive–compulsive.

^a^Not significant anymore when controlling for age and gender.

^b^Significant when controlling for age and gender.

*⁣* ^*∗*^ When two random effects are listed (separated by “|”) it indicates two nested random intercepts, one for participants and one for days within each participant.

## Data Availability

The data that support the findings of this study, corresponding analysis code, and EMA items are openly available in OSF at https://osf.io/fr5b4.

## Endnotes

^1^However, we acknowledge that putatively maladaptive regulation strategies may not always influence the outcome negatively and vice versa, putatively adaptive regulation strategies may not always be beneficial. Indeed, ER results depend on multiple factors [68].

^2^Analyses were conducted with and without the first 10 participants missing the affect item disgusted, which did not change the significance of the vast majority of our findings. The results reported herein include these 10 participants.

^3^Please note for models with ER effectivity as outcome variable that even though median ICC suggested a three-level structure, models did not converge anymore when we assumed a three-level structure and included additional random slopes (due to the low number of observations per participant and day). We therefore only included two nested random intercepts for this outcome variable.

^4^Please note that we treated avoidance-oriented and engagement-oriented ER strategies as continuous variables because the data and most importantly, the residuals of our fitted models were approximately normally distributed. A Q–Q plot of model residuals can be found in Figure B.1 of the supporting information.

^5^Please note that a particularly pronounced floor effect for EC or other ER measures in the OCD sample cannot explain our non-significant findings. Descriptive statistics do not indicate extremely restricted endorsement patterns (Table 2).

## Supporting Information

Supporting Information In the Supporting Information, we report estimates of multilevel models including the outcome variable's score from the previous assessment point as additional control variable (Table A.1). Including this control variable was part of our preregistration but we had to change our data analytic procedure to maintain statistical power. We also show Q–Q plots (Figure B.1) for residuals of models with avoidance-oriented or engagement-oriented ER strategies as outcome variable to demonstrate normal distribution. In addition, we provide a table with between-person and within-person correlations for all EMA variables of interest (Table C.1). We also include an interaction plot (Figure D.1) when predicting engagement-oriented ER strategies for readers who are interested in the interpretation of this effect. Lastly, we provide equations for all multilevel models for interested readers and upon reviewer's request.
