# Supplementary material for: Associations Among in-The-Moment Emotional Clarity, Emotion Regulation, and Psychopathology in Obsessive-Compulsive Disorder
Source: Depress Anxiety. 2025 Nov 14;2025:7799020. doi: 10.1155/da/7799020 (PMC12638152; doi:10.1155/da/7799020)
Supplement: Supporting Information — In the Supporting Information, we report estimates of multilevel models including the outcome variable's score from the previous assessment point as additional control variable (Table A.1). Including this control variable was part of our preregistration but we had to change our data analytic procedure to maintain statistical power. We also show Q–Q plots (Figure B.1) for residuals of models with avoidance-oriented or engagement-oriented ER strategies as outcome variable to demonstrate normal distribution. In addition, we provide a table with between-person and within-person correlations for all EMA variables of interest (Table C.1). We also include an interaction plot (Figure D.1) when predicting engagement-oriented ER strategies for readers who are interested in the interpretation of this effect. Lastly, we provide equations for all multilevel models for interested readers and upon reviewer's request. [file 7799020.f1.pdf]

Supplements

Table A.1

Estimates for multilevel regression models assessing associations between emotion regulation behavior and emotional clarity (with lagged outcome variable as additional control variable)

	Estimate	*z-value*	*p-value*	.95 CI	Random effect (*SD*)
A. Current ER Effectivity (*D* = 42.9%)[1]					
**Intercept**	**51.088**	**11.788**	**<.001**	**42.593; 59.582**	5.711
Current EC	1.332	0.434	.664	-4.678; 7.343	
**Group**	**-6.333**	**-1.997**	**.046**	**-12.547; -0.119**	
Current EC:Group	-0.509	-0.157	.875	-6.872; 5.854	
**Mean EC**	**7.803**	**2.225**	**.026**	**0.929; 14.677**	
**Mean EC:Group**	**-9.418**	**-2.464**	**.014**	**-16.908; -1.927**	
**Current Negative Affect**	**-10.817**	**-6.540**	**<.001**	**-14.059; -7.575**	
**Lagged ER Effectivity**	**0.180**	**3.197**	**.001**	**0.070; 0.290**	
B. Current No. of AO Strategies (*D* = 72.2%)					
**Intercept**	**1.445**	**5.253**	**<.001**	**0.906; 1.985**	0.646
**Current EC**	**-0.644**	**-2.544**	**.011**	**-1.139; -0.148**	
**Group**	**0.705**	**2.457**	**.014**	**0.143; 1.268**	
Current EC:Group	0.499	1.887	.059	-0.019; 1.018	
Mean EC	-0.550	-1.330	.183	-1.359; 0.260	
Mean EC:Group	0.730	1.626	.104	-0.150; 1.610	
Current Negative Affect	0.272	1.734	.083	-0.035; 0.579	0.533
**Lagged No. of AO Strategies**	**0.254**	**5.453**	**<.001**	**0.163; 0.345**	
C. Current No. of EO Strategies (*D* = 55.4%)					
**Intercept**	**1.553**	**5.338**	**<.001**	**0.983; 2.124**	0.705
**Current EC**	**0.836**	**3.120**	**.002**	**0.311; 1.362**	
Group	0.092	0.295	.768	-0.517; 0.700	
**Current EC:Group**	**-0.710**	**-2.521**	**.012**	**-1.262; -0.158**	
Mean EC	0.323	0.731	.465	-0.544; 1.189	
Mean EC:Group	-0.0002	0.000	.999	-0.945; 0.944	
Current Negative Affect	0.163	1.151	.250	-0.114; 0.439	0.276
Lagged No. of EO Strategies	0.092	1.652	.099	-0.017; 0.200	

Note. EC = emotional clarity; ER = emotion regulation; AO = avoidance-oriented; EO = engagement-oriented; OC = obsessive-compulsive; *D* = explained Deviance. Significant estimates are written in bold font. [1] due to the low number of observations it was not possible to test a three-level structure for this model.

Figure B.1

Q-Q plots for residuals of models with avoidance-oriented or engagement-oriented ER strategies as outcome variable

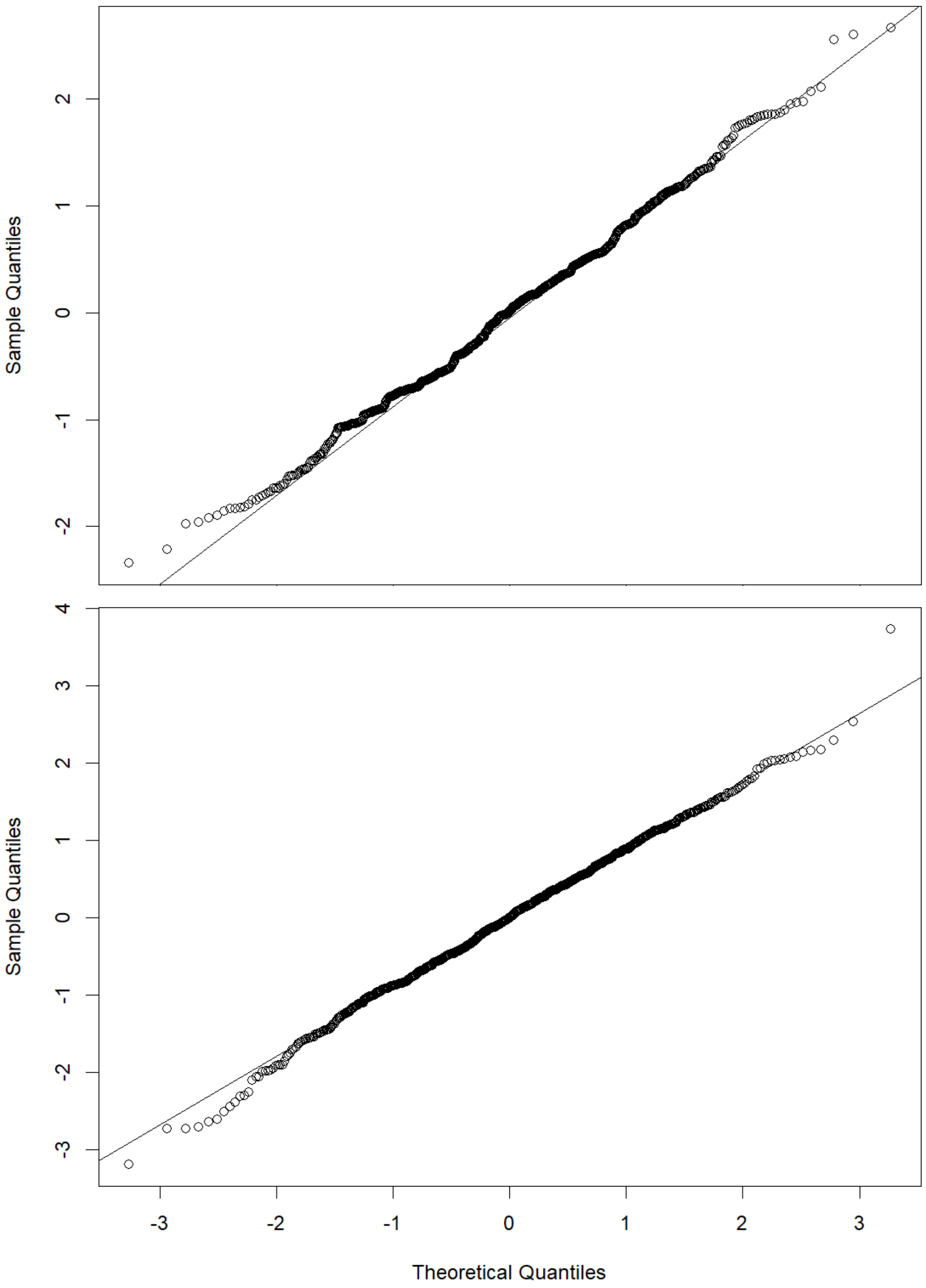

Note. Plots indicate that model residuals are approximately normally distributed.

Table C.1

Correlation matrix

Variable	1	2	3	4	5
1. Emotional Clarity	--	-.12**	.13**	.17**	-.07**
2. Number of Avoidance-oriented ER Strategies	-.17	--	-.24**	-.17**	.17**
3. Number of Engagement-oriented ER Strategies	.29**	-.15	--	.27**	<.01
4. Self-perceived ER Effectiveness	.33**	-.50**	.32**	--	-.12*
5. Presence of OC Symptoms[1]	-.06	.39**	-.02	-.12	--

Note. Within-person correlations on a momentary level are shown above the diagonal and between-person correlations on an aggregated trait level are shown below the diagonal. [1] = All correlations with OC symptoms are reported for individuals of the OCD group only. ER = emotion regulation; OC = obsessive-compulsive. * $p < .05$ ** $p < .01$.

Figure D.1

Plot for the marginally significant interaction effect between momentary emotional clarity and group membership when predicting currently used number of engagement-oriented ER strategies

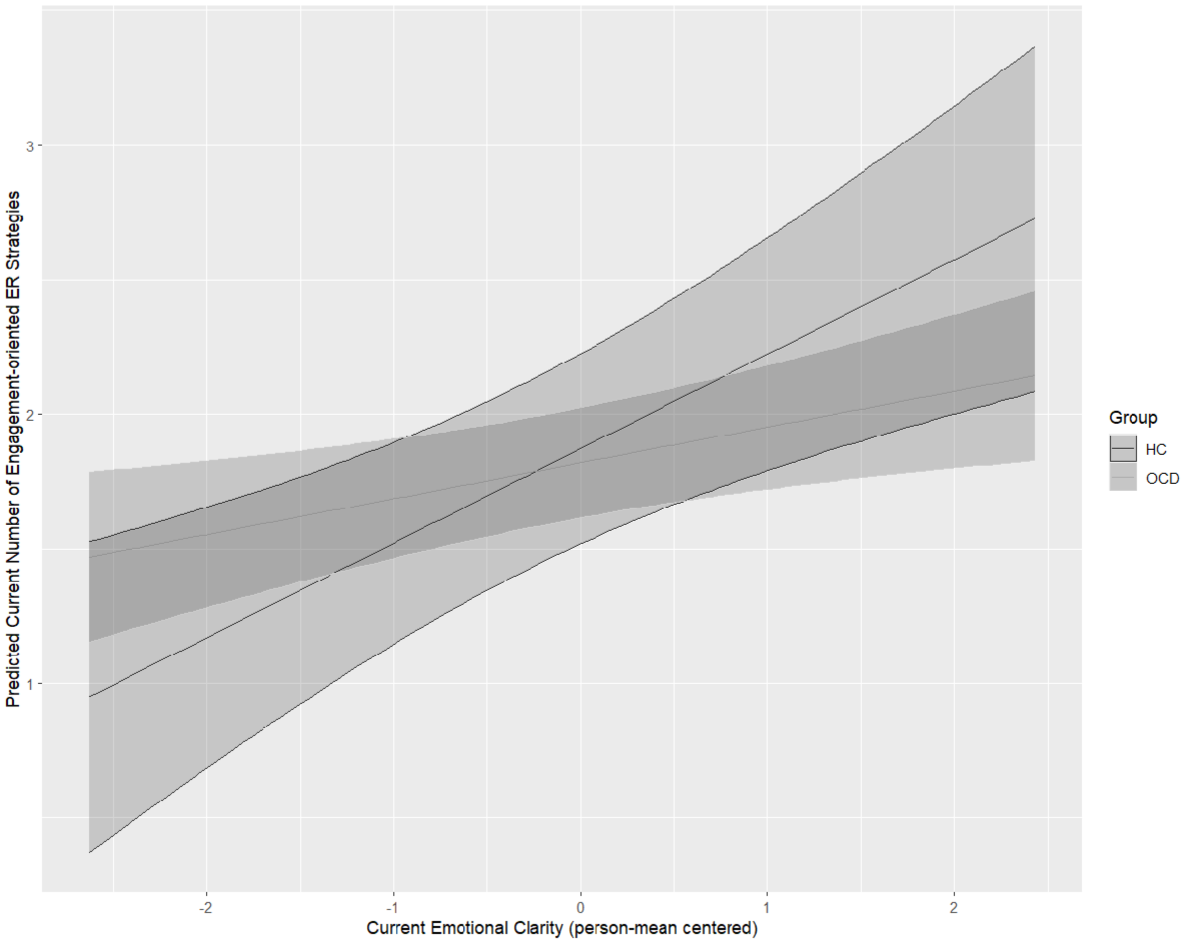

Note. ER = emotion regulation; HC = mentally healthy controls; OCD = individuals with obsessive-compulsive disorder.

Multilevel model equations

We tested hypothesis 1 with the following multilevel model:

$Yikj$(Emotional clarity on time point k for person i) = ($\beta 0$+ $b0i$) +

$\beta 1$*(group) + $\beta 2$* (baseline depression) + $\beta 3$*(age) + $\beta 4$*(gender) + εijk

For the assessment of hypothesis 2, we used the following multilevel models:

$Yijk$(ER effectivity on time point k for person i *on day* j) = ($\beta 0$+ $b0i$+ $b0ij$) +

$\beta 1$*(emotional clarity on time point k) + $\beta 2$* (mean negative affect on time point k) +

$\beta 3$*(person mean of emotional clarity) + $\beta 4$*(group) + $\beta 5$*(emotional clarity on time point k*group) + $\beta 6$*(person mean of emotional clarity*group) + $\beta 7$*(age) +

$\beta 8$*(gender) + εijk

$Yijk$(Used number of avoidance-oriented ER strategies on time point k for person i) =

($\beta 0$+ $b0i$) + $\beta 1$*(emotional clarity on time point k) + ($\beta 2$+ $b2i$) * (mean negative affect on time point k) + $\beta 3$*(person mean of emotional clarity) + $\beta 4$*(group) +

$\beta 5$*(emotional clarity on time point k*group) + $\beta 6$*(person mean of emotional clarity*group) + $\beta 7$*(age) + $\beta 8$*(gender) + εijk

$Yijk$(Used number of engagement-oriented ER strategies on time point k for person i) =

($\beta 0$+ $b0i$) + $\beta 1$*(emotional clarity on time point k) + ($\beta 2$+ $b2i$) * (mean negative affect on time point k) + $\beta 3$*(person mean of emotional clarity) + $\beta 4$*(group) +

$\beta 5$*(emotional clarity on time point k*group) + $\beta 6$*(person mean of emotional clarity*group) + $\beta 7$*(age) + $\beta 8$*(gender) + εijk

We tested hypothesis 3 with the following multilevel models:

$Yijk$(Presence of OC symptoms on time point k for person i on day j) = ($\beta 0$+ $b0i$+ $b0ij$) +

($\beta 1$ + $b1i$)*(emotional clarity on time point k) + $\beta 2$*(presence of OC symptoms on time point k-1) + $\beta 3$*(person mean of emotional clarity) + $\beta 4$*(age) +

$\beta 5$*(gender) + εijk

Y_{ijk}(Presence of OC symptoms on time point $k+1$ for person i on day j) = ($\beta_0 + b_{0i} + b_{0ij}$) + β_1*(emotional clarity on time point k) + β_2*(presence of OC symptoms on time point k) + β_3*(person mean of emotional clarity) + β_4*(age) + β_5*(gender) + ε_{ijk}

Y_{ijk}(Emotional clarity on time point k for person i on day j) = ($\beta_0 + b_{0i} + b_{0ij}$) + ($\beta_1 + b_{1i}$) * (presence of OC symptoms on time point k) + β_2*(emotional clarity on time point $k-1$) + β_3*(person mean of OC symptom frequency) + β_4*(age) + β_5*(gender) + ε_{ijk}

Y_{ijk}(Emotional clarity on time point $k+1$ for person i on day j) = ($\beta_0 + b_{0i} + b_{0ij}$) + β_1*(presence of OC symptoms on time point k) + ($\beta_2 + b_{2i}$) * (emotional clarity on time point k) + β_3*(person mean of OC symptom frequency) + β_4*(age) + β_5*(gender) + ε_{ijk}
